# Comparison of Artificial and Spiking Neural Networks on Digital Hardware

**DOI:** 10.3389/fnins.2021.651141

**Published:** 2021-04-06

**Authors:** Simon Davidson, Steve B. Furber

**Affiliations:** APT Group, Department of Computer Science, University of Manchester, Manchester, United Kingdom

**Keywords:** artificial neural network, spiking neural network, deep neural network, rate-based encoding, neuromorphic hardware

## Abstract

Despite the success of Deep Neural Networks—a type of Artificial Neural Network (ANN)—in problem domains such as image recognition and speech processing, the energy and processing demands during both training and deployment are growing at an unsustainable rate in the push for greater accuracy. There is a temptation to look for radical new approaches to these applications, and one such approach is the notion that replacing the abstract neuron used in most deep networks with a more biologically-plausible spiking neuron might lead to savings in both energy and resource cost. The most common spiking networks use *rate-coded* neurons for which a simple translation from a pre-trained ANN to an equivalent spike-based network (SNN) is readily achievable. But does the spike-based network offer an improvement of energy efficiency over the original deep network? In this work, we consider the digital implementations of the core steps in an ANN and the equivalent steps in a *rate-coded* spiking neural network. We establish a simple method of assessing the relative advantages of rate-based spike encoding over a conventional ANN model. Assuming identical underlying silicon technology we show that most rate-coded spiking network implementations will not be more energy or resource efficient than the original ANN, concluding that more imaginative uses of spikes are required to displace conventional ANNs as the dominant computing framework for neural computation.

## 1. Introduction

Within the broad field of Artificial Neural Networks (ANNs) the development of Deep Neural Networks (DNNs) over the last decade has made a number of significant applications possible (Graves et al., 2013; Barsoum et al., 2016; Howard et al., 2017; Vinyals et al., 2019), elevating the neural network from a laboratory-bound curiosity to a dependable tool for real world applications in the areas of image and speech recognition (Graves et al., 2013; Kepuska and Bohouta, 2018; Brown et al., 2020). The focus of much recent research has been classification accuracy while other considerations such as the energy and computational cost during both training and deployment have arguably been of secondary interest. Each year brings new progress—with higher recall accuracy on industry standard benchmarks—but also brings with it greater computational, energy, and data storage demands (Han et al., 2015; Strubell et al., 2019). Researchers in both industry and academia are now searching for disruptive new approaches to avoid this barrier to future progress.

One approach is to go back to neuroscience. Deep neural networks are—after all—highly abstracted from the biological networks in Nature. While real neurons communicate using spikes of potential, sent between neurons via a web of connections, in ANNs the output of each artificial neural unit is a multi-valued *activation* often described as a proxy for the firing rate of the true neuron, or as a measure of the mean activity of a group of neurons (Averbeck et al., 2006). Research into Spike-based Neural Networks (SNNs) has continued in academia but is only recently received much attention from industry (such as the Intel Loihi; Davies et al., 2018) for a variety of reasons: the lack of a robust learning rule for spiking networks comparable with the backpropagation learning rule in ANNs; the immaturity of rules for the design and composability of networks of spiking neurons in contrast to the established frameworks for ANNs such as TensorFlow (Abadi et al., 2015); and the lack of a compelling demonstrator for spiking networks. With this renewed focus on spiking neurons it is timely to re-examine the capabilities of spike-based networks in the domains currently dominated by Deep ANNs. Do they offer potential savings in energy or computational cost over ANNs for the same work done?

In this paper we examine this possibility with respect to the *rate-coded* spiking network, a commonly used neural architecture in which information is transmitted through the *rate* at which a neuron produces spikes and for which the precise time of individual spikes has no significance. Such networks are of interest in part because a spiking equivalent can be readily obtained from a pre-trained ANN (Rueckauer et al., 2017) and in part because the output of a neuron can easily be interpreted by an external observer (for example, a higher spike rate indicates a higher degree of belief in the presence of a particular feature in the input). In this work we restrict our focus to digital implementations of such networks.

By comparing the digital implementations of the core computations for both the conventional neural network and the spike-based equivalent, under the assumption of identical silicon substrates, we show that most rate-coded spiking network implementations will **not** compete with the ANN.

The structure of the paper is as follows. We present a brief overview of the key processes in a deep neural networks (section 2), then present two implementations of these processes: one as a conventional ANN and the other as a spiking neural network (section 3). Our key evidence is presented as an elaboration of the computational steps of these circuits for a standard silicon process (section 4) before concluding (section 5).

## 2. Overview of Network Structure and Computations

**Figure 1:**
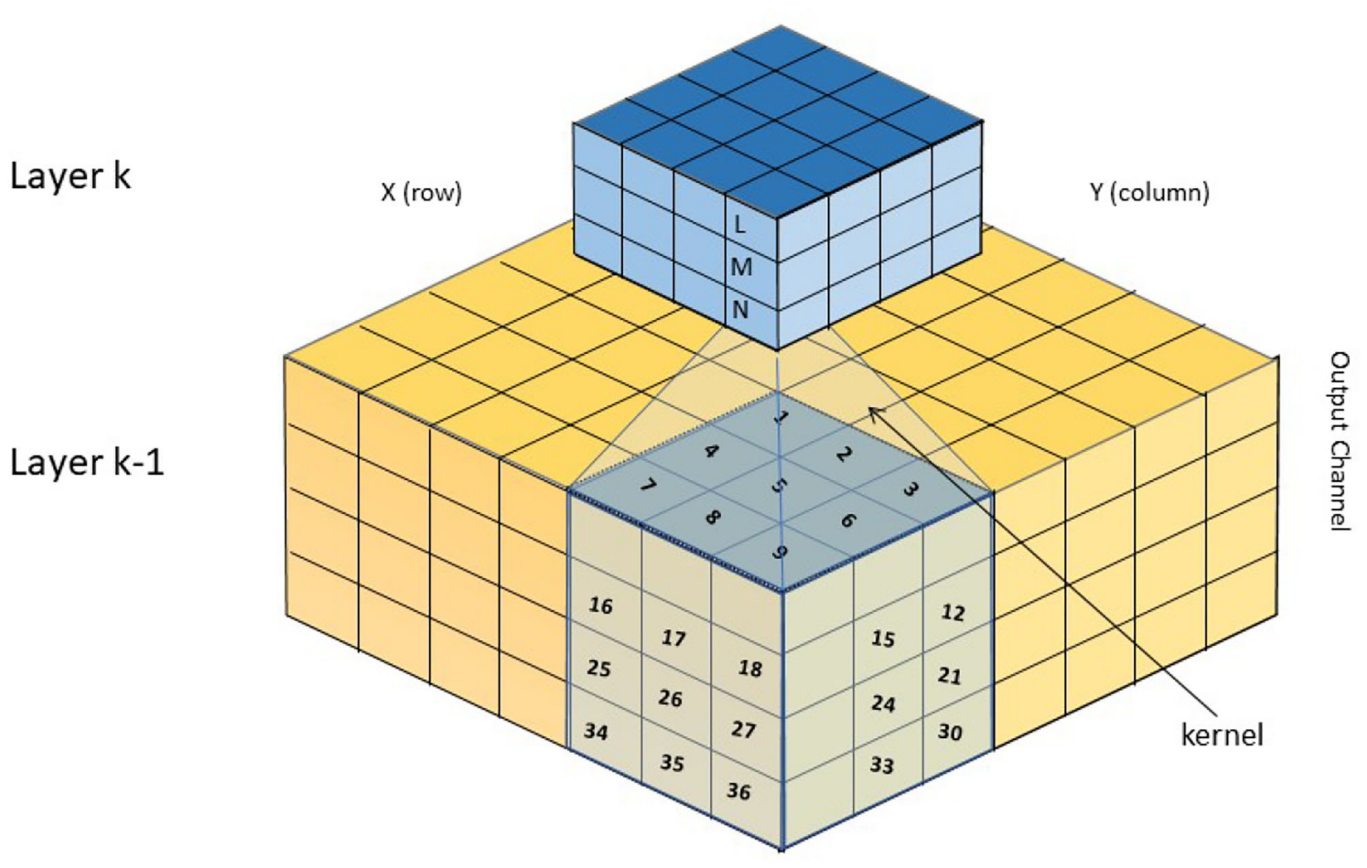
Schematic of a convolutional neural network, showing two layers. Each layer consists of multiple 2-D planes of neurons, one layer per output channel (feature). A given neuron on layer k receives input from a patch of the layer below, each input weighted by a set of kernel coefficients. The same set of coefficients is typically shared by every neuron on the same layer, although different output channels have distinct kernels.

A Deep Neural Network consists of multiple layers of artificial neurons with more than one hidden layer between unique layers receiving input and producing output, respectively. Layers are connected in a stack with the output of one connecting forward to one (and occasionally more) of the layers higher in the stack (Liu et al., 2017; Alom et al., 2019). Input is presented at the lowest layer and activity propagates up to the highest layer, where output activity can be decoded (Figure 1). Layers typically implement a *convolution kernel* with each neuron in a layer receiving the activations from a patch of the neurons in the layer below. The neuron performs a weighted sum of these input activations weighted by the kernel coefficients and this sum passes through an output non-linearity to produce its own activation value, to be passed upwards.

Many layers in a DNN implement this weighted sum operation, while others implement *normalization* and *pooling* operations, which are computationally much less demanding (Howard et al., 2017). We focus our attention on the basic convolutional layer, which consumes the majority of the resources.

Processing for a single neuron in a convolutional layer of an ANN consists of the following steps:

- Input handling: Receiving incoming activity from neurons in the layer(s) below.
- Kernel coefficient retrieval: For each incoming activation, obtain the value of the strength of the connection from the source neuron to each target neuron in the layer.
- Processing: For each target neuron update an accumulator with the product of the incoming activation and the associated synaptic weight.
- Threshold and output: Once all inputs have been processed, perform the non-linear threshold operation (typically ReLU) on each neuron and transmit the output activation values to the next layer(s).

For convolutional layers the set of synaptic weights forming one kernel may be shared by all of the neurons in the layer (LeCun et al., 2015). Typically, for the upper layers of the stack weight sharing is no longer possible as neurons specialize but the size of these layers is usually much smaller than the lowest layers (Howard et al., 2017).

As noted earlier, it is straightforward to translate a deep neural network into an equivalent rate-encoded spiking neural network and there are open-source tools available to do this (Rueckauer et al., 2017). Such a conversion process leads to a network with a one-to-one correspondence both between the neurons in the two networks and with regards to the inter-neuron connections. A given activation value in the ANN domain then maps to a number of spikes in the spiking domain. From this simplified overview we can now consider the form of a digital implementation of the key processing steps in both the ANN and the SNN.

## 3. Implementations of the ANN and SNN Versions of Key Processes

In this section we describe the implementations of the ANN and SNN in standard digital hardware based on how this is done in software on SpiNNaker, the million core neural simulation platform developed at The University of Manchester (Furber et al., 2014; Rhodes et al., 2018). From the literature describing other implementations, such as the Intel Loihi (Davies et al., 2018; Lines et al., 2018), we expect these basic steps to be representative of other implementations.

**Figure 2:**
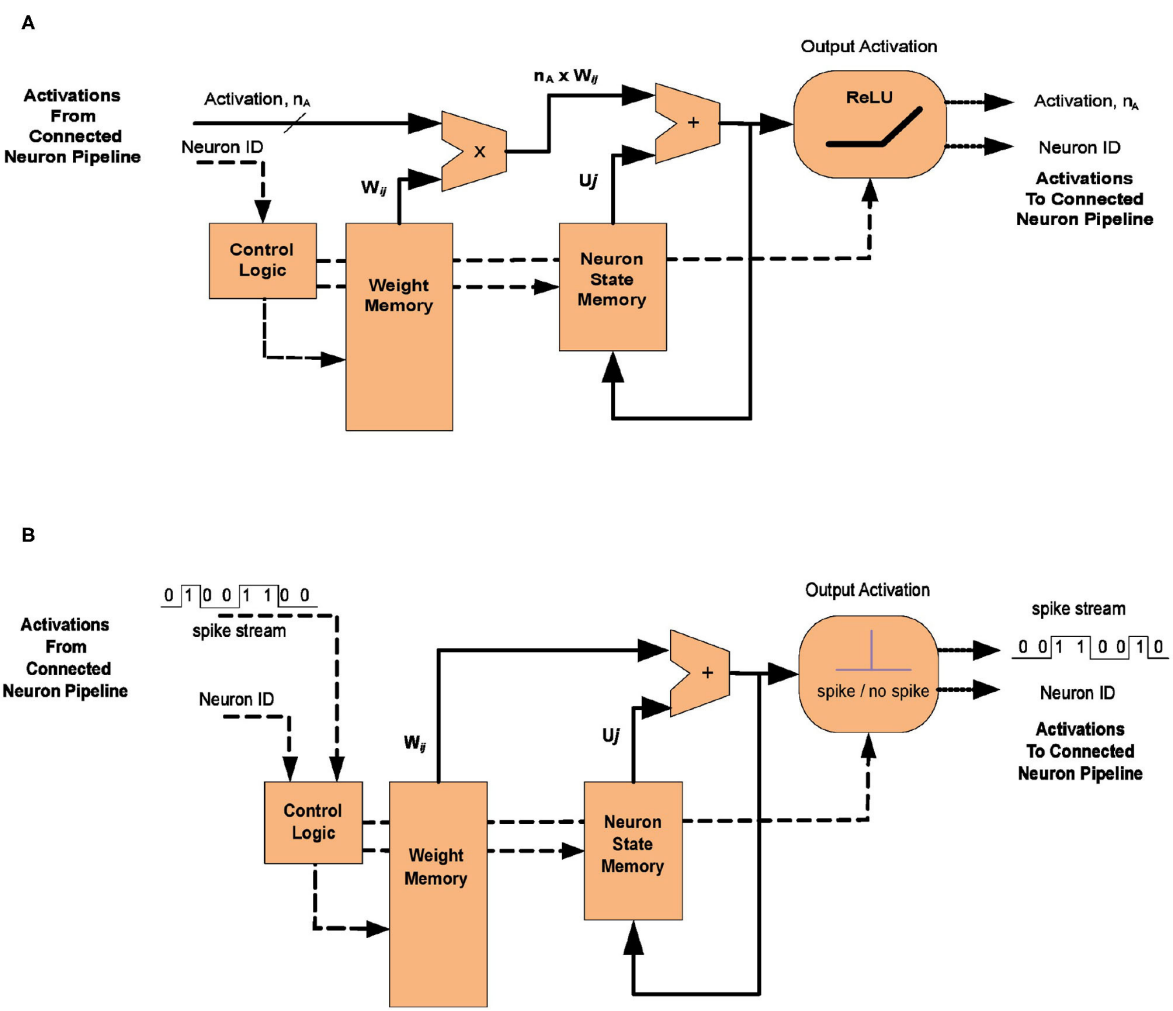
**(A)** One possible implementation of the neuron update pipeline for a conventional Artificial Neural Network (ANN). In the ANN the activation is a multi-bit value (typically FLOAT16 and even INT8 in some recent hardware) which must be multiplied by the connection weight, *W*_*ij*_ before being added to the soma potential (or accumulator value), *U*_*j*_. **(B)** In the equivalent SNN implementation the neuron output is a single spike whose arrival causes the associated weight *W*_*ij*_ to be added directly to the potential *U*_*j*_ and no multiplication is necessary.

As noted in section 2, it is convenient to view the principal steps on the neural algorithm beginning with the arrival of a spike or activation from a source neuron arriving at a target neuron. In a digital implementation of neural networks it is normal to map a group of neurons to a single processing module or pipeline, the neural state being updated sequentially using shared resources. This is appropriate when the speed of execution of the module (of the order of GigaHertz in a modern process) greatly exceeds the arrival rate of incoming activations (of the order of one every one tenth of a millisecond), allowing the efficiency gains that can be obtained from the time-domain multiplexing of shared hardware. Figure 2 shows a generic implementation of both an Artificial Neural network (Figure 2A) and its spiking equivalent (Figure 2B). The operations in the ANN are described first, for hardware representing a group of neurons in a convolutional layer of the network.

An activation arrives from a neuron in the layer below. This information consists of an identifier of the neuron, *X*_*i*_, and its activation value. The identifier may be implicit if activations arrive in a prescribed order. The activation value is conveyed using a bus consisting of *n*_*a*_ bits. Fewer bits require less energy to transmit and use, but reduces the information content. Recent work with deep networks has considered the trade-off of range versus performance, leading to GPUs providing support for INT8 (8-bit) values (NVIDIA, 2019).

The incoming activation will come from a source neuron in the receptive field of one or more of the neurons managed by this neuron processor. While for the purposes of illustration we normally consider the fan-in of one neuron through its kernel connections, for the implementation the arrival of an activation triggers the update of neurons to which it connects and so a fan-out organization of data is more appropriate. To process each affected connection, we require two pieces of state:

- Current accumulator value of the target neuron, *U*_*j*_.
- Kernel co-efficient, representing the strength of connection from the source to the target neuron, *W*_*ij*_.

Each neuron has a state variable, the accumulator, representing the cumulative input it has received on this pass through the network. The number of state bits per neuron is a function of the number of bits in the connection weight and the fan-in of the target neuron. It will typically be less than 32-bit and so for a few hundred neurons it is practical to keep this information local to the neuron pipeline in a small register file or RAM.

Since an incoming activation will fan-out to many target neurons, an associative memory can be used to map the ID of the incoming activation to a list of its targets. Each entry in the list consists of:

- The ID of the target neuron, *Y*_*j*_.
- the weight strength, *W*_*ij*_, of the connection.

Parsing this list item by item, the target neuron ID *Y*_*j*_ is used to read the corresponding accumulator value, *U*_*j*_, from the neuron state memory and the weight strength from the weight memory; the weight strength is multiplied by the incoming activation value to produce a signed product; the accumulator is summed with the resulting signed product; the new accumulator is written back to the neuron state memory. Each accumulator update is therefore a multiply-accumulate (MAC) operation and there is one for each target neuron.

The multiplier used to generate the product of the activation and the weight would be implemented using a variant of Booth encoding, which yields a fast, efficient circuit (He and Chang, 2008). The energy dissipated in each use of the multiplier is a function of the bit patterns. Smaller values of the activation would lead to fewer transitions in the multiplier logic, lower energy usage and hence a more efficient processor. We return to this in section 4.

Once all incoming activations have been processed, the resulting accumulators are passed through a thresholding function. The most commonly used is ReLU, whose output is defined as *max*(*x, 0*) for input *x*.

Each new activation is sent to the interconnect fabric for transmission to neuron units managing the next layer of the network.

Now consider the equivalent functionality in the implementation of a spiking neural network made up of Leaky-Integrate-and-Fire (LIF) neurons, shown in Figure 2B. The input coming from another neuron is now a stream of spikes that capture the same information as the activation in the ANN.

Each incoming spike must now trigger the reading of the same associative memory defined for the ANN, using the ID *X*_*i*_ as the key, retrieving the list of synaptic weights *W*_*ij*_ and their corresponding target neuron IDs, *Y*_*j*_. Each entry in the list will trigger the reading of the neural state *U*_*J*_ of a target neuron, given its identifier *Y*_*J*_.

Where the functionality of the SNN begins to diverge from that of the ANN is that no multiplication is required at this point. The synaptic weight *W*_*ij*_ is merely added to the current state *U*_*j*_, which can then be written back to the state memory. If the neuron state has surpassed the pre-defined threshold then an output spike is generated and the neuron state value is reset (either to an absolute value or by a fixed amount) before it is written back (Rueckauer et al., 2017).

## 4. Analysis of Computational and Energy Costs for The ANN and the SNN

We now quantify the energy consumption in the two network types (ANN and SNN), again focusing on the processing of pre-synaptic input arriving at a neuroprocessor pipeline responsible for a group of neurons.

For the ANN, the following energy costs are required to process the input activation arriving from a single neuron:

- Transmission cost of the activation to this neuroprocessor, *E*_*sendActivation*_.
- For each target neuron:
  - Retrieval of one item from a list of synaptic connections, from local weight memory, *E*_*retrieveWeight*_.
  - Retrieve neural state for that neuron from state memory, *E*_*getState*_.
  - Multiply activation by weight, *E*_*multiply*_.
  - Add product to the neuron state, *E*_*stateAdd*_.
  - Writeback neuron state to state memory, *E*_*writeState*_.

The total energy consumed depends on the number of resident target neurons, *N*_*meanTargets*_ to which the pre-synaptic neuron connects. The size, *N*_*activationBits*_ and number of set bits, *N*_*setBits*_ in the activation will also affect the energy consumed in the multiplier, which would typically be Booth encoded (He and Chang, 2008).

Thus we can express the total energy, *E*_*totalANN*_, consumed in the processing of a single incoming activation as:

(1) $$\begin{array}{l} E_{totalANN}=E_{broadcastActivation}\;+\;E_{retrieveWeights}\;+\\ \;N_{meanTargets}\times [E_{getState}+E_{multiplication}+E_{Addition}\\ \;+E_{writeState}] \end{array}$$

The read, multiply, and writeback operations each consume about 300 fJ and these occur for each target neuron in the pipeline.

For the spiking network the cost is the same per incoming event except that there is no multiplication step and the initial energy cost of transmitting the spike, *E*_*broadcastSpike*_, will be lower since it requires fewer bits. However, the entire cost of processing this event will occur for *each* spike. If the mean number of spikes required to represent an activation is *N*_*meanSpikes*_, then the total energy cost per received neuron output for the spiking network, *E*_*totalSNN*_, is:

(2) $$\begin{array}{l} E_{totalSNN}=N_{meanSpikes}\times (E_{broadcastSpike}+E_{retrieveWeights})\\ \;+N_{meanSpikes}\times N_{meanTargets}\times (E_{getState}+E_{Addition}\\ \;+E_{writeState}) \end{array}$$

To quantify the two models we draw on energy consumption estimates from post-layout analysis of the data for the SpiNNaker2 development using TSMC 22FDX technology assuming average process at room temperature (Höppner et al., 2019; Höppner et al., 2020). All energy estimates take account of both dynamic switching power and leakage. A single 32-bit read access to 2K word SRAM costs 296 fJ, while a 32 × 8 bit multiply-accumulate costs 293 fJ. We will assume that the write back cost to the SRAM is approximately the same as the read and that a 32-bit addition costs one fifth the cost of the MAC operation. To simplify the discussion, let E represent the adder energy cost (approx 60 fJ), thus the multiply costs 5E, SRAM read 5E, and SRAM writeback E. We shall use the SRAM cost for both weight and neuron state accesses.

The broadcast energy will be highly dependent on the implementation. We will neglect it for this reason, which benefits the SNN over the ANN since this cost is borne more times in the former than in the latter.

In the ANN case the weight retrieval costs 5E and the loop over each target costs 5E + 5E + E + E = 12E. This cost occurs *N*_*meanTargets*_ times.

In the SNN case, the loop for each arriving spike over each target costs 5E + E + E = 7E. This cost also occurs *N*_*meanTargets*_ times for each arriving spike.

For the SNN energy to be lower than the ANN for this technology would require the expected number of spikes used to transmit an output to be <12E/7E or 1.72. At this low level of activity, the coding of outputs using spikes is arguably no longer a rate-based code. This is our result.

## 5. Discussion

The argument presented above focuses on the fundamental computation in the ANN and SNN cases. It suggests that only networks with very low spiking activity justify the use of spikes over conventional ANNs. A crucial factor in this result is the cost of accessing both neuron state and the weight matrix. These energy costs typically exceed the computational cost and must be taken into consideration when assessing the efficiency of the encoding mechanism for output values. While acknowledging that variations in process technologies will have some impact on the relative costs of memory, adders and multipliers, these variations will be small and the essential conclusion should not change significantly. The design is free to modify the parameters of our proposed metric for their technology.

Does our result imply that spiking networks implemented on digital hardware have no potential benefit? We believe that the opposite is true, but success or failure is strongly linked to the choice of encoding. We have demonstrated that to beat an conventional ANN representation requires sparse and/or temporally-coded vectors of spiking neurons for which the presence of a spike carries more information than is achieved with rate-coded networks in which spike rates per neuron measured in the tens or even hundreds of Hertz are the norm. The intent of this work is to focus on more information rich coding schemes, which necessitate moving away from an SNN as merely a re-coding of an equivalent ANN.

Such approaches do exist and we wish to encourage others to adopt the same mindset. A group of researchers at TU Graz demonstrated an image recognition network in which each neuron produces at most one spike (Stöckl and Maass, 2021), while also proposing another distinct encoding using two spikes per neuron (Stöckl and Maass, 2020). Very sparse codes have been demonstrated as more energy efficient than equivalent ANN networks, even with spike rates as low as 0.24 spikes per neuron to perform an inference (Lee et al., 2019; Wu J. et al., 2019; Wu Y. et al., 2019). FPGA-based circuits using very sparse encoding combine good accuracy with low power when applied to classification tasks (Mostafa et al., 2017). Another slightly older line of enquiry is the use of *rank-order coding*, a form of temporal encoding that may be relevant to understanding how the retina encodes information (Thorpe and Gautrais, 1998; Furber et al., 2007; Portelli et al., 2016).

These examples illustrate that more efficient encoding methodologies are readily achievable. They have the potential to avoid the inefficiencies we have outlined in the implementation of rate-coded networks. The path towards truly mould-breaking neurally-inspired computation for artificial systems should focus on information encoding as the way forward.

## Data Availability Statement

The original contributions presented in the study are included in the article/supplementary material, further inquiries can be directed to the corresponding author/s.

## Author Contributions

SD wrote the paper, with feedback from SF. All authors contributed to the article and approved the submitted version.

## Conflict of Interest

The authors declare that the research was conducted in the absence of any commercial or financial relationships that could be construed as a potential conflict of interest.
